# Lysosome Function and Dysfunction in Hereditary Spastic Paraplegias

**DOI:** 10.3390/brainsci11020152

**Published:** 2021-01-24

**Authors:** Daisy Edmison, Luyu Wang, Swetha Gowrishankar

**Affiliations:** Department of Anatomy and Cell Biology, College of Medicine, University of Illinois at Chicago, Chicago, IL 60612, USA; dedmis2@uic.edu (D.E.); lwang240@uic.edu (L.W.)

**Keywords:** lysosome, HSP, motor neurons, Alzheimer’s, axon

## Abstract

Hereditary Spastic Paraplegias (HSPs) are a genetically diverse group of inherited neurological diseases with over 80 associated gene loci. Over the last decade, research into mechanisms underlying HSPs has led to an emerging interest in lysosome dysfunction. In this review, we highlight the different classes of HSPs that have been linked to lysosome defects: (1) a subset of complex HSPs where mutations in lysosomal genes are causally linked to the diseases, (2) other complex HSPs where mutation in genes encoding membrane trafficking adaptors lead to lysosomal defects, and (3) a subset of HSPs where mutations affect genes encoding proteins whose function is primarily linked to a different cellular component or organelle such as microtubule severing and Endoplasmic Reticulum-shaping, while also altering to lysosomes. Interestingly, aberrant axonal lysosomes, associated with the latter two subsets of HSPs, are a key feature observed in other neurodegenerative diseases such as Alzheimer’s disease. We discuss how altered lysosome function and trafficking may be a critical contributor to HSP pathology and highlight the need for examining these features in the cortico-spinal motor neurons of HSP mutant models.

## 1. Introduction

Hereditary Spastic Paraplegias (HSPs) are among the most genetically diverse inherited neurological diseases, with over 80 disease loci identified to date [1]. Despite the genetic heterogeneity, certain common cellular mechanistic themes underly HSP dysfunction [2]. Research, especially over the last decade, has revealed that several HSPs are associated with abnormalities in the endo-lysosomal system (see Table 1). The endo-lysosomal pathway and closely related autophagic pathway are responsible for protein and organelle turnover in all cells, including neurons. In addition to HSPs, dysfunction in the autophagic and lysosomal pathways have been linked to many other neurodegenerative diseases, such as Alzheimer’s disease (AD) and Parkinson’s disease [3]. So far, HSPs identified to be associated with lysosomal defects can be classified into three main categories. Category 1 is a subset of complex HSPs with autosomal recessive inheritance where the causal mutation is in genes encoding lysosomal proteins. These are characterized by progressive spastic paraplegia along with thinning of the corpus callosum, white matter abnormalities, and cognitive impairment. Examples include SPG11 and 15, which are discussed in depth in this review (also see Table 1). Category 2 involves complex HSPs where the associated genes do not directly encode lysosomal proteins, but rather proteins involved in membrane trafficking whose loss of function appears to also lead to defects in lysosomes. This includes HSPs associated with mutations in genes encoding the Adaptor protein-4 (AP-4) complex. We will discuss our own novel findings supporting AP-4 mediated regulation of neuronal lysosome traffic. The last category (Category 3; Table 1) involves HSPs where the mutant proteins have well-established roles in other pathways and at other organelles. This includes mutations in a gene encoding microtubule severing protein, Spastin, whose deregulation is the most common cause of HSP. Recent studies have also reported lysosome dysfunction in neurons carrying mutations in *spast*. However, in all of these cases, the precise nature of lysosome dysfunction in HSP and its relevance to disease pathology is not well understood. We will discuss the current understanding of lysosomal traffic and function in these different contexts, including our findings with regards to the AP-4 complex. We also highlight similarities in axonal lysosome pathology observed in several HSPs and Alzheimer’s disease. Lastly, we examine the lysosomal heterogeneity in response to HSP mutations and speculate on axonal lysosomes as a point of vulnerability in HSP pathology.

## 2. SPG11 and SPG15

Mutations in the spastic paraplegia genes *SPG11*, encoding spatacsin, and *ZFYVE26,* encoding Spastizin (SPG15), cause the most prevalent forms of autosomal-recessive hereditary spastic paraplegia with thinning of the corpus callosum (AR-HSP-TCC) [46,47,48,49]. The clinical phenotype of SPG11 and SPG15 are nearly indistinguishable [47], and are characterized by walking difficulties, progressive spasticity, cognitive impairment, thin corpus callosum, and white matter abnormalities [50]. Magnetic Resonance Imaging (MRI) of SPG15 affected siblings revealed marked thinning of corpus callosum and the “ears of the lynx” sign noted in many SPG11 and SPG15 patients [5,47,51].

Spatacsin is encoded by the *SPG11* gene, which contains 40 exons on chromosome 15q21.1. [49]. The *SPG15* causal gene locus was refined to a 2.64 Mb genetic interval on chromosome 14q23.3–q24.2. [50,52]. *ZFYVE26* mRNA has been shown to be widely distributed in human tissues and rat embryos. In adult rodent brains, its RNA expression profile closely resembles that of SPG11 [50].

Spastizin contains a FYVE domain [50], which is a highly conserved zinc-finger-binding domain. Many FYVE-finger proteins interact with different forms of phosphoinositides. Spastizin’s FYVE domain binds to phosphatidylinositol 3-phosphate (PtdIns3P/PI3P) in vitro [4], which is normally highly enriched in early endosomes and autophagosomes. Zfyve26 protein is broadly expressed in the mouse brain [53]. At the subcellular level, several studies initially reported Spastizin localizing to the endoplasmic reticulum and early endosomes [49,50,53,54,55]. However, subsequent studies have largely established them to be late endosomal and lysosomal in localization [4,7]. In HeLa cells, HA-tagged and endogenous SPG15 localized predominantly to puncta labeled by markers for lysosomes and minimally with early endosome markers [7].

**Figure 1:**
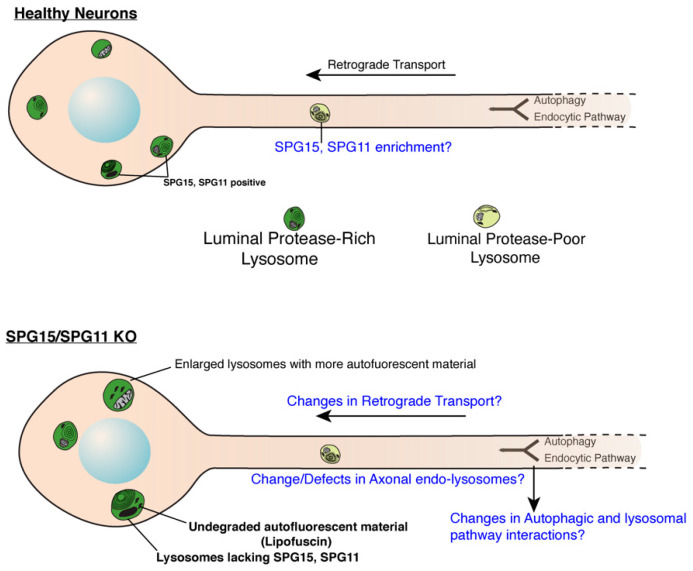
**Role of SPG11 and SPG15 in regulating endo-lysosomes in neurons.** Cartoon showing endo-lysosomes in healthy or SPG11/SPG15 KO neurons. SPG11 and 15 localize to late endosomes and lysosomes in soma of healthy neurons while they are lacking in the KO neurons. Lysosomes in KO neurons are enlarged and accumulate autofluorescent, undegraded material called lipofuscin. It remains to be investigated if SPG11/15 localize to axonal lysosomes even in healthy neurons. Likewise, effect of SPG11/15 loss on aspects of axonal lysosomes (such as their transport, composition and interaction with autophagosomes) remains to be investigated. Open questions are indicated in blue (Cartoon modified from Gowrishankar et al., PNAS, 2015) [56].

In SPG11 and SPG15 patient fibroblast, HeLa, and hTERT-RPE1 cells depleted of SPG11 or SPG15 by siRNA, organelles enriched in LAMP1 (Lysosome associated Membrane Protein1) were significantly enlarged as compared with controls without changes in total LAMP1 levels [4,5]. LAMP1 is highly enriched on late endosomes and lysosomes, thus implicating SPG11 and SPG15 as regulators of lysosome morphology (Figure 1). Loss of SPG11 causes cholesterol accumulation in lysosomes as well as a concurrent increase in cytosolic calcium due to altered store-operated calcium entry [6]. Much like human SPG15 patients, SPG15 KO mice exhibit progressive spastic gait disorder with cerebellar symptoms and degeneration of both motor cortex neurons and cerebellar purkinje cells [53]. Interestingly, prior to degeneration, neurons showed accumulations of large autofluorescent deposits intensely labeled by the lysosomal marker LAMP1 [53]. This suggests a link between lysosome dysfunction and the development of neurodegenerative symptoms. SPG15 patient fibroblasts also exhibit accumulation of immature autophagosomes and increased MAP1LC3B-II and SQSTM1/p62 levels: results that are replicable in SPG15-depleted HeLa cells and murine hippocampal neurons [55]. This suggests an additional SPG15-related autophagic defect, although dysfunctional lysosomes could account for this as well.

Varga et al. generated a model of SPG11 where they inactivated *spg11* by inserting a gene-trap cassette in the first intron of the gene [57]. Branchu et al. (2017) developed a second mouse model of SPG11 that encapsulated the key features of SPG11 patients [58]. This mouse model is characterized by motor deficits that develop much earlier than the *spg11*-knockout mouse developed by Varga et al. It also presents with degeneration of lower motor neurons, thinning of the corpus callosum, and cognitive impairment: characteristics that are absent in the Varga et al. (2015) model [57,58]. The differences observed between the two mouse models could be a result of the strategies used to inactivate *spg11.* Varga et al. inactivated *spg11* by inserting a gene-trap cassette in the first intron of the gene [57], whereas Branchu et al. inserted stop codons in exon 32 to mimic the most frequent mutation observed in *SPG11* patients [49,58].

Although there are apparent discrepancies with regard to the phenotypes expressed by each mouse model, the cellular alterations seem to be related. Loss of Spatacsin/SPG11 in the mouse model generated by Varga and colleagues exhibited accumulation of lipids in lysosomes, suggesting that SPG11 plays a role in lysosomal function via regulation of lipid homeostasis. 

Chang et al. implicated both SPG11 and SPG15 in the crucial process of autophagic lysosome reformation (ALR). ALR is a model for lysosome reformation from autolysosomes and is the last step of the autophagy process [4], a phenomenon needed to generate “new” lysosomes for future fusion events. This is required following the formation of autolysosomes: the fusion products of autophagosomes and lysosomes. After autophagic material has been degraded, proto-lysosomes extrude from autolysosomes and mature into functional lysosomes, thus restoring lysosomal balance in the cell. Loss of SPG11 or SPG15 results in depletion of “free” lysosomes (available for future fusion events) and autolysosome accumulation, suggesting ALR failure. SPG11 and SPG15 were also shown to be essential components in the initiation of autolysosomal tubulation [4]. Thus, the proposed mechanism potentially links SPG11 and SPG15 to autophagy/lysosomal biogenesis machinery and neurodegeneration [4]. Further analysis of SPG11 and SPG15 in the context of neuronal ALR is still required, but it will be interesting to examine whether ALR is indeed altered in SPG11 and 15 KO neurons (Figure 1). Studies that focus on axonal autophagosomes and lysosomes will be of particular interest, as it has been suggested that consumption of axon-generated autolysosomes is dependent on retrograde trafficking to the soma [59,60,61]. This would suggest any ALR in neurons may primarily occur in soma and not in axons once autophagic cargo is degraded.

Although SPG11 and 15 loss of function (LOF) studies show clear involvement in neuropathology, studies examining their localization and effect on lysosome function in cortico-spinal motor neurons (CSMN) will be critical to elucidating patho-mechanisms underlying SPG11 and SPG15-linked HSP.

## 3. AP-5 Complex

Adaptor proteins that usually contain a variety of protein binding motifs link protein binding partners together and facilitate assembly of larger complexes. The AP-5 complex is the most recently discovered of the five adaptor complexes, which bears very little sequence similarity to the remaining four [62]. It is a low-abundance complex composed of four subunits, ζ and β5 (large subunits), μ5 (medium subunit), and σ5 (small subunit). It is the least conserved of the complexes across species and is absent in several model organisms. Expression of GFP-tagged AP-5 revealed that some of the puncta colocalized with LAMP1, indicating late endosomal and lysosomal localization [63]. Gene expression profile of AP-5 (http://biogps.org/) supports a role across different regions of the brain, including tissues that are relevant to the pathogenicity of HSP, such as the spinal cord.

While some initial studies suggested possible DNA helicase activity for the ζ subunit of AP-5, this has since been disproven [62]. siRNA experiments targeting AP-5 ζ have implicated the complex in endo-lysosomal trafficking. Loss of any of the subunits appears to cause accumulation of early endosomal compartments devoid of internal vesicles but enriched with cation-independent mannose-6-phosphate receptor (CI-M6PR) and retromer complex components, Vps35 and Vps29. This phenotype is also observed upon knockdown of SPG11 and 15. All four subunits of AP-5, along with SPG11 and 15 have been demonstrated to form a hetero-hexameric complex that is localized to late endosomes and lysosomes [7], potentially via interaction of the FYVE domain of SPG15 with Pi3P on the organelles. CRISPR-mediated KO of AP-5 ζ, followed by sub-cellular fractionation and mass spectrometric analysis has revealed CI-M6PR, as well as Golgi proteins GOLIM4 (golgi integral membrane protein 4) and GOLM1 (golgi membrane protein 1), as AP-5 cargo; retrieval of these proteins from late endosomes to the trans-golgi network is inhibited upon loss of AP-5 complex function [64]. Currently, only the AP-5 ζ subunit has been assigned an HSP number (SPG48) [65]. Clinical features of SPG48 patients encompass prominent spastic paraparesis, sensory and motor neuropathy, ataxia, dystonia, myoclonus, and parkinsonism. Skin fibroblasts from these patients also revealed accumulation of material in multi-lamellar organelles, indicating a lysosomal storage defect [8].

Given the phenotypic similarity of SPG11/15/AP-5 loss with regard to endo-lysosomes, this further cements links between dysfunction in lysosomal pathways and HSP pathology.

## 4. AP-4 Complex

AP-4 is a hetero-tetrameric adaptor protein that is ubiquitously expressed in low abundance in humans [66,67]. It was discovered by the Robinson and Bonifacino groups due to its sequence similarity to other adaptor protein (AP) family members [66,67]. To date, of the five known AP complexes, AP-4 is one of the least understood [62]. However, recent observations from five independent studies in human patients have linked mutations in all four AP-4 subunits (μ1; AP4M1(SPG50), β1; AP4B1(SPG47), ε1; AP4E1(SPG51), and σ1; AP4S1(SPG52)) to progressive Spastic Paraplegia, a disease characterized by a complex phenotype that manifests in infancy or early childhood [22,62,68,69]. Core clinical features include hypotonia, cognitive and motor delays, seizures, facial dimorphism, stereotypic laughter with tongue protrusion, and thinning of the corpus callosum [68,70]. In fact, the phenotype is robust enough to be considered a clinically recognizable “AP-4 deficiency syndrome.” The disease symptoms also highlight that AP-4 is an obligatory hetero-tetramer, where loss of any of the subunits is sufficient to cause loss of complex functionality. Due to this disease connection, understanding the mechanism of neuronal AP-4 function is of great interest and relevance to the HSP field.

Immuno-electron microscopy studies have localized AP-4 to a perinuclear trans-golgi network compartment. Current evidence supports a role for AP-4 in promoting autophagosome biogenesis through regulation of autophagy-related protein 9 (ATG9) export from the trans-golgi network to autophagosomes [15,19,20,21]. ATG9 is the only transmembrane protein of the core autophagy machinery, and its distribution is heavily dependent on vesicular trafficking [71,72,73]. Consequently, loss of AP-4 function leads to retention of ATG9 in the trans-golgi network and defective autophagosome generation [15]. This phenotype has been observed in AP-4 ε KO mice, patient-derived fibroblasts, and iPSC-derived neurons [15,18,19,20].

**Figure 2:**
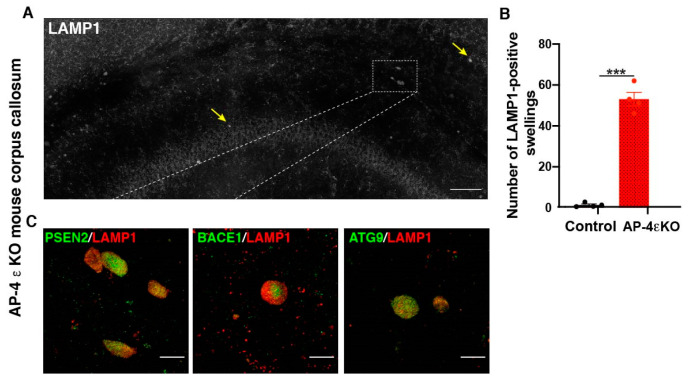
**Accumulation of autophagic and lysosomal organelles enriched in APP-processing machinery in dystrophic axons in AP-4 ε KO mice** (**A**) Stitched image of a portion of AP-4 ε KO mouse cerebral cortex stained for LAMP1, showing several LAMP1-filled axonal dystrophies in corpus callosum (CC) (yellow arrows; white box). (**B**) Quantification of LAMP1-filled axonal dystrophies in the CC per animal. n = 4 animals per genotype (sex-matched littermate controls were used). (**C**) Enrichment of PSEN2, BACE1, and ATG9 in axonal swellings. Scale bar, 50 μ (**A**) and 10 μ (**C**). ***- (*p* < 0.001, unpaired *t* test).

In addition to alterations in ATG9 traffic and autophagosome maturation, AP-4 appears to play a role in lysosome biogenesis and traffic as well. Intriguingly, loss of AP-4 affects lysosomes differently in the axon compared to the soma. Previous studies have demonstrated that AP-4 ε KO mice exhibit axonal swellings in various regions of the brain and spinal cord [20]. The axonal swellings in hippocampus and white matter tracts of the midbrain were found to be enriched in the late endosomal and lysosomal protein, LAMP1. Our lab has carried out a systematic analysis of the organelles accumulating in swollen axons in AP-4 ε KO mouse brain, focusing on the dystrophies in the corpus callosum (Figure 2A). We find that there is indeed a dramatic accumulation of LAMP1-positive organelles in axonal swellings (Figure 2A,B). We also find that ATG9 positive vesicles are enriched in these swellings (Figure 2C). While this result seems surprising, given ATG9 export out of trans-golgi network is hampered, we speculate that lack of sufficient ATG9 on the axonal autophagosomes prevents their subsequent maturation (and possibly, their interaction with the endo-lysosomes) and transport out of axons. Thus, over time, large numbers of ATG9-positive organelles build up in AP-4 ε KO axons [20]. Super-resolution imaging and examination of ATG9- and LAMP1-double positive and single-positive vesicles is needed to determine relative abundance of autophagosomes and autolysosomes here and if there are autophagosome maturation defects as well as stalling of autolysosomes. At this point, based on our data we can state that lysosome and autophagic intermediates build up in axonal swellings in these neurons while no such buildup is observed in the soma. Indeed, AP-4 has been implicated in polarized sorting, supporting the differential effect in distinct subcellular compartments, upon its loss [74]. Our detailed characterization of this phenotype has revealed that this bears striking similarity to what is observed in human Alzheimer’s brain tissue and mouse models of the disease [56,75,76,77,78]. This is discussed in greater detail in Section 7, *‘Links between HSPs and Alzheimer’s disease’.*

Current mouse models have greatly advanced our understanding of cellular functions of AP-4, but the relatively mild phenotypes seen in these mice (such as hind-limb clasping defects hinting at motor-neuronal defects) stand in stark contrast to human disease symptoms. This suggests an additional, non-redundant role for human AP-4. Studies in human stem cell-derived neurons could thus be invaluable in furthering our understanding of AP-4. Likewise, studies in CSMN in AP-4 mice may shed light on lysosomal traffic and function in these disease-relevant neurons.

## 5. Spastin

Autosomal dominant mutations in the gene that encodes Spastin (SPG4), a microtubule severing ATPase, are the most common cause of HSP [2]. An interesting study has linked Spastin’s microtubule severing function with Endoplasmic Reticulum-mediated endosome tubule fission and lysosome function [28]. The authors demonstrated that Spastin (a microtubule regulator) and endosomal protein IST1 localize to Endoplasmic Reticulum-endosome contacts and play a critical role in efficient severing of endosomal tubules. This defective endosome fission leads to altered trafficking of CI-M6PR, aberrant delivery of lysosomal enzymes, and abnormal lysosome morphology in mutant primary neurons as well as human stem cell-derived neurons deficient in Spastin. Thus, they are responsible for cargo-sorting through the process of endosome tubule fission [28]. While *spastin* mutant neurons exhibit enlarged lysosomes in their soma, they also exhibit a strong axonal lysosome phenotype [28]. Axonal swellings in primary cortical neurons carrying a *spastin* mutation exhibit accumulation of LAMP1 positive vesicles. This phenotype is highly reminiscent of the lysosome-filled axonal dystrophies observed in Alzheimer’s disease. These also bear striking similarity to the dystrophies we observed and characterized in AP-4 ε KO mice. It remains to be seen if the nature and composition of these LAMP1 vesicles is the same in these different conditions.

Intriguingly, cellular models lacking Strumpellin (a component of the WASH complex) or Endoplasmic Reticulum-shaping protein REEP1 show similar endosome tubule fission and lysosome abnormalities [28]. Mutations in both of these cases also cause HSP. Thus, the relationship between Endoplasmic Reticulum-endosomal fission and lysosomal function appears to link different “classes” of HSP proteins to a unifying pathway of axon degeneration. 

## 6. JIP3/MAPK8ip3

JIP3/MAPK8iP3 and its orthologs in nematode worms and zebrafish have been implicated in retrograde axonal lysosome transport [79,80,81,82]. In mammalian systems (mice and iPSC-derived neurons), loss of JIP3 leads to axonal swellings that are filled with lysosomes [81,82]. In the last two to three years, recurrent de novo variants of mapk8ip3 have been identified in individuals who manifest with mild to severe intellectual disability, hypoplasia of corpus callosum, and cerebral or cerebellar atrophy [83]. In two of these cases, spasticity was also reported. Expression of these variants in *c elegans* leads to increased density of axonal lysosomes, implicating axonal lysosome traffic in neuropathology. Importantly, in addition to the aforementioned symptoms, a second study reporting *mapk8ip3* variants in four families described spastic diplegia as a major symptom [84]. This reiterates the importance of axonal lysosome traffic in neurological and neurodegenerative diseases.

## 7. Links between HSPs and Alzheimer’s Disease

**Figure 3:**
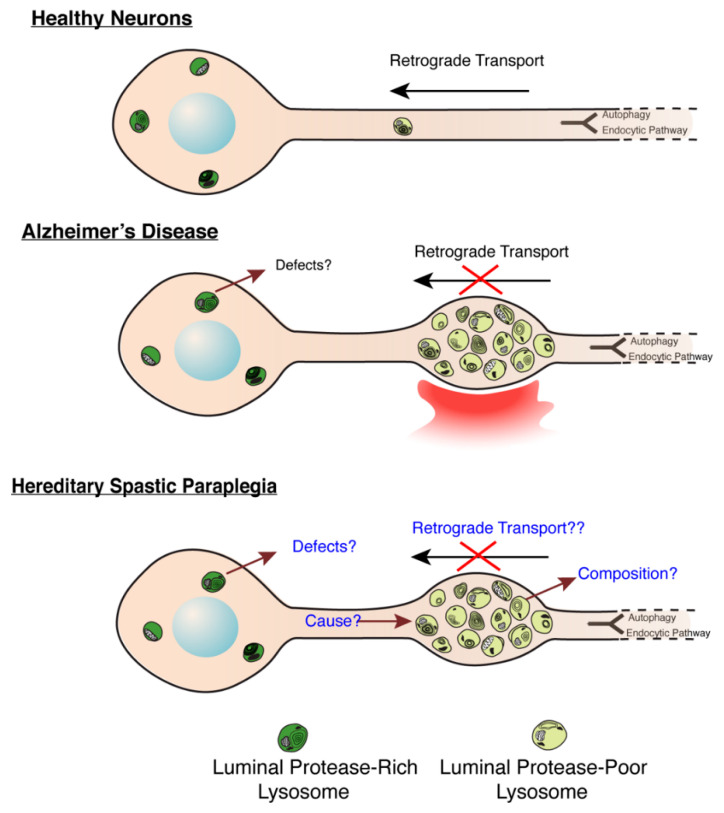
**Molecular Mechanisms underlying lysosome dysfunction in HSP and AD**. (Modified from Gowrishankar et al., PNAS, 2015) [56]. Build-up of lysosome-like organelles (derived from a mix of endocytic and autophagic pathways, depicted in schematic to feed into common pathway) in axons in AD and HSP. In healthy and disease states, lysosomal organelles in axons are deficient in luminal proteases, while those in soma are enriched in these proteases. Thus, accumulation of protease-poor lysosomes in axons will have consequences on protein and organelle turnover in this compartment. In AD, a signal from a plaque potentially induces a defect in axonal lysosome transport. In HSPs with this axonal lysosome build-up, we propose that the underlying cause for axonal lysosome build-up is the failure of trafficking of a critical regulator of axonal lysosome transport to these organelles.

**Figure 4:**
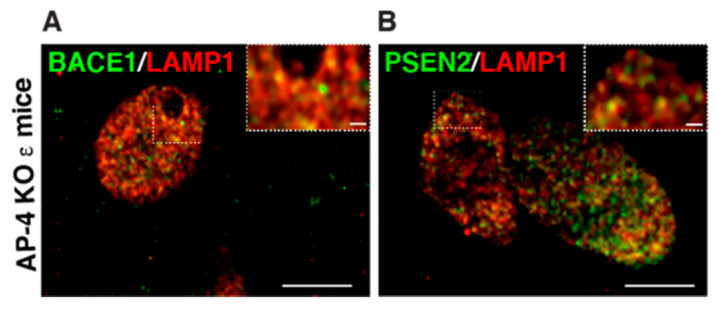
**Examination of BACE1 and PSEN localization in axonal dystrophies of AP-4 ε KO mice, at high resolution** (**A**,**B**) Representative airyscan images of LAMP1 with BACE1 (**A**) and PSEN2 (**B**) showing most BACE1 and PSEN2 vesicles in the axonal swellings are also LAMP1-positve, unlike what is observed with ATG9 and LAMP1. Scale bar: 5 μ; inset 1 μ.

The axonal swellings filled with LAMP1-postive vesicles observed in AP-4 ε KO and *spastin* mutant neurons are highly reminiscent of the lysosomes that build up in dystrophic axons of amyloid plaques in Alzheimer’s disease [56,75,76,77,78] (Figure 3). These accumulating axonal lysosomes have been implicated as sites of Aβ production in AD, and thus contribute to disease progression. Such sites are enriched in amyloid precursor protein (APP), as well as APP processing enzymes: BACE1 (β-site APP cleaving enzyme) and PSEN2 (presenilin 2) [56,76]. Given the similarity of axonal lysosome build up in AP-4 ε KO brains, we examined the distribution of BACE1 and PSEN2 in them. Indeed, we found BACE1 and PSEN2 strongly accumulate in the swollen axons of AP-4 ε KO mouse brains (Figure 2C). BACE1 and PSEN2 accumulation in LAMP1-positive organelles was confirmed using airyscan imaging (Figure 4A,B).

**Figure 5:**
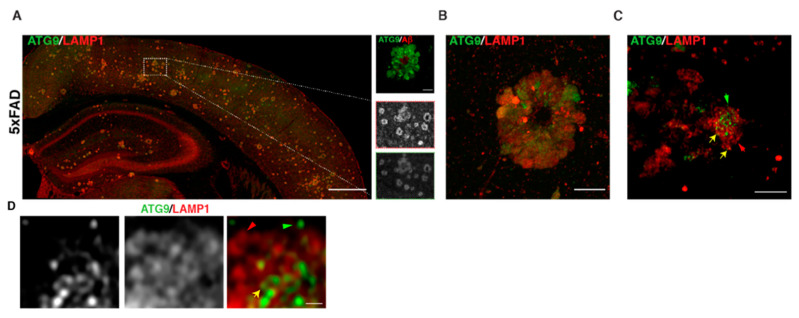
**Autophagic and lysosomal organelles building up in swollen axons in AD mouse brain includes ATG9-positive vesicles** A–D. ATG9 and LAMP1 staining. (**A**) Stitched image of 5 × FAD mouse cerebral cortex showing accumulation of ATG9 and LAMP1 in axonal swellings around plaques. Region within white box is zoomed in the inset. (**B**) Standard confocal image of one amyloid plaque in 5 × FAD, which hints at varying levels of ATG9 vesicles in different dystrophies. (**C**) Airyscan image of a selection of dystrophies at one plaque shows that distinct vesicles that are positive for only LAMP1 (red arrow) or ATG9 (green arrow) or double positive (yellow arrow) are discernable. (**D**) Zoomed in look at a portion of one axonal swelling (airyscan mode) showing distinct vesicles for the first time. Scale bars—200 μ (**A**), 10 μ (**B**), 5 μ (**C**), and 1 μ (**D**).

Given the similarities in axonal buildup of organelles in AP-4 ε KO to those in AD (including enrichment of BACE1 and PSEN2), we tested if ATG9 was enriched in dystrophic axons in AD plaques. Indeed, we observed a robust accumulation of ATG9 in swollen axons of amyloid plaques (Figure 5A–D) in 6-month-old 5 × FAD mice, a transgenic mouse model that robustly develops amyloid plaque pathology [85]. Consistent with our findings, a study published in late 2019 demonstrated that ATG9 is enriched in dystrophies around plaques in two different AD mouse models and is in fact one of the earliest proteins to build up in these dystrophies [86]. This reiterates the need to better understand the maturation, transport, and consumption of axonal ATG9-positive vesicles, as this could shed additional light on mechanisms underlying AD and HSP pathology. Conducting AP-4 LOF studies in AD mice would help elucidate whether AP-4 mediated regulation of axonal lysosomes is a critical part of AD pathology. Conversely, it would be interesting to determine if there are defects or changes relating to AP-4 complex levels and/or function in AD mouse models. Experiments addressing how AP-4 haploinsufficiency affects AD pathology in mice will carry important implications for human heterozygotes with AP-4 mutations, especially with regard to developing Alzheimer’s disease. It also raises the question of whether HSP patients, where HSP mutations are linked to axonal lysosome defects, have an increased risk of developing AD. Indeed, two independent studies have described unusual mutations in Presenilin 1 in several members of a Greek [87] and Finnish family [88] with Early Onset Familial Alzheimer’s disease where these individuals developed spastic paraparesis prior to occurrence of dementia. Conversely, dementia and cognitive impairment has been associated with certain cases of complex HSPs [89,90,91]. Examining HSP mouse models for AD-related pathology could shed further insight on this topic.

Thus, understanding of lysosome function, especially axonal lysosome traffic and function, will be critical to understanding mechanisms of HSP pathology. While we have focused on neuronal lysosomes, it is possible that defects in lysosomes in astrocytes and glia also contribute to HSP pathology. Studies examining lysosomal biogenesis and trafficking in these cell types in HSP models will shed light on this. Given the growing evidence linking lysosome dysfunction to HSP, we propose that alterations in endo-lysosomal function in motor neurons, especially of those in axons, are a critical contributor to HSP pathogenesis. This will need to be tested by examining lysosomal transport and function in cortico-spinal motor neurons in HSP mouse models and/or motor neurons differentiated from human stem cells. These insights could also prove to be invaluable to our understanding of mechanisms underlying other neurodegenerative diseases.

## Figures and Tables

**Table 1:** **HSP genes, categorized by function**.

Category	Gene	Protein Name	Endo-Lysosomal Defect	Type, Age of Onset and Clinical Features	Reference
**1**	*SPG11*	Spatacsin	Reduced Autophagic lysosomal reformation Defective lysosomal degradation and morphology Cholesterol accumulation in lysosomes	AR Complex Early onset Walking difficulties, progressive spasticity, cognitive impairment, thin corpus callosum, white matter abnormalities, retinal abnormalities, parkinsonism	[4,5,6]
	*SPG15 (ZFYVE26)*	Spastizin (ZFYV26 or FYVE-CENT)	Reduced Autophagic lysosomal reformation Defective lysosomal degradation and morphology	AR Complex Early onset Walking difficulties, progressive spasticity, cognitive impairment, thin corpus callosum, white matter abnormalities, retinal abnormalities, parkinsonism	[4,5,7]
	*SPG48 (AP5Z1)*	AP5Z1	Altered late endosome to golgi trafficking	AR Complex Later onset than SPG11 and 15 Prominent spastic paraparesis, sensory/motor neuropathy, ataxia, dystonia, myoclonus, parkinsonism	[8,9]
**2**	*SPG21*	Maspardin	Altered EGF signaling (loss of Maspardin affects growth and maturation of neurons)	AR Complex Late adolescent to early adult onset Dementia, cerebellar and extrapyramidal signs, thin corpus callosum, white matter abnormalities (Mast syndrome)	[10,11]
	*SPG33 (ZFYVE27/Protrudin)*	ZFYVE27	Implicated in intracellular trafficking in the corticospinal tract; Defective neurite extension	AD Pure Late onset	[12,13,14]
	*SPG47 (AP4B1)*	AP4B1	Defective autophagosome maturation	AR Complex Early onset severe intellectual disability, microcephaly, seizures, and growth retardation, thin corpus callosum	[15,16]
	*SPG50 (AP4M1)*	AP4M1	Defective autophagosome maturation and glutamate receptor trafficking	AR Complex Early onset severe intellectual disability, microcephaly, seizures, and growth retardation, thin corpus callosum	[16,17,18]
	*SPG51 (AP4E1)*	AP4E1	Defective autophagosome maturation	AR Complex Early onset severe intellectual disability, microcephaly, seizures, and growth retardation, thin corpus callosum	[19,20,21]
	*SPG52 (AP4S1)*	AP4S1	Defective autophagosome maturation	AR Complex Early onset severe intellectual disability, microcephaly, seizures, and growth retardation, thin corpus callosum	[16,22]
**3**	*SPG3A*	Atlastin-1 (Interacts with Spastin)	Impaired BMP signaling Impaired formation and function of Endoplasmic Reticulum/Golgi due to its role in membrane tubulation/vesiculation	AD Pure Early onset	[23,24,25]
	*SPG4 (SPAST)*	Spastin	Diminished microtubule severing (including in the context of endosomal tubulation/fission) Endoplasmic Reticulum morphogenesis (interactions with reticulons, atlastin, REEP1) Endoplasmic Reticulum -mediated endosomal tubule fission defects; altered endosomal traffic Defective endosomal sorting of certain proteins such as M6PR. Altered BMP signaling, Cytokinesis; Axonal lysosome build-up	AD Pure Typically, early onset; some cases are late onset	[26,27,28]
	*SPG6 (NIPA1)*	NIPA1	Downregulation of BMP receptors by promoting their endocytosis and lysosomal degradation Accumulation of tubulovesicular organelles with endosomal features at axonal and dendritic terminals	AD Pure Late adolescent to early adult onset	[29,30,31]
	*SPG8 (KIAA0196)*	Strumpellin	Endosomal morphogenesis Cytoskeletal regulation Decreased abundance of CAV1 protein Endoplasmic Reticulum -mediated endosomal tubule fission defects; altered endosomal traffic Defective endosomal sorting of certain proteins such as M6PR.	AD Pure Adult onset	[28,32,33]
	*SPG10 (KIF5A)*	KIF5A	Axonal transport defect of mitochondria. Defects in autophagic flux, lysosome function on perturbation of KIF5a	AD Usually pure but complex in certain cases Pure onset: Can be early (infancy) or adult onset (30 years) Complex features: peripheral neuropathy, severe upper limb amyotrophy, mental impairment, parkinsonism, deafness, retinitis pigmentosa	[34,35,36,37]
	*SPG20*	Spartin	Altered endosomal traffic and BMP signaling Lipid droplet biogenesis Decreased Microtubule stability regulation via the BMP-dFMRP-Futsch pathway (role in synaptic growth, neuronal survival)	AR Complex Dysarthria, progressive lower extremity spasticity and weakness, distal muscle wasting (Troyer syndrome), developmental delay, short stature, subtle skeletal abnormalities	[38,39]
	*SPG31 (REEP1)*	REEP1	Disrupted Endoplasmic Reticulum -mitochondria contacts Endoplasmic Reticulum membrane curvature induction/Endoplasmic Reticulum- shaping defects Endoplasmic Reticulum-mediated endosomal tubule fission defects; altered endosomal traffic and defective endosomal sorting of certain proteins such as M6PR.	AD Pure and Complex Bimodal onset before 20 and after 30 (late onset) Complex phenotype: axonal peripheral neuropathy, cerebellar ataxia, tremor, dementia	[28,40,41,42]
	*BICD2*	BICD2	Increased binding affinity for dynein-dynactin motor complex and sequesters RAB6, impairing neurite growth	AD Complex Typically early onset; some cases are late onset Myopathy with slow progression	[43]
	*DNM2*	Dynamin 2	Endosomal trafficking dysfunction as well as altered phagosome maturation.	AD Pure Adult onset	[44]
	*TUBB4A*	TUBB4A	Impaired binding of motor protein to microtubules stunts neurite outgrowth Possible disruption of mitochondrial transport	AD Complex developmental delay, dystonia, choreoathetosis, ataxia, pyramidal and cerebellar features, hypomyelination and atrophy of the cerebellum or basal ganglia	[45]

AD: Autosomal Dominant; AR: Autosomal Recessive; Pure: Spasticity alone; Complex: includes other neurological features in addition to spasticity.

## Data Availability

The data presented in this study are available on request from the corresponding author.

## Abbreviations

**Acronym**	**Denotation**
AP	Adaptor protein
AP-4	Adaptor protein-4
AP-5	Adaptor protein-5
AD	Alzheimer’s disease
APP	amyloid precursor protein
ALR	autophagic lysosome reformation
ATG9	autophagy-related protein 9
AR-HSP-TCC	autosomal-recessive hereditary spastic paraplegia with thinning of the corpus callosum
CI-M6PR	cation-independent mannose-6-phosphate receptor
CSMN	cortico-spinal motor neurons
HSPs	Hereditary Spastic Paraplegias
LAMP1	Lysosome associated Membrane Protein1
LOF	Loss of function
MRI	Magnetic Resonance Imaging
PtdIns3P/PI3P	phosphatidylinositol 3-phosphate
PSEN2	presenilin 2
TCC	thin corpus callosum
FYVE domain	Fab1, YOTB, Vac1, and EEA1 domain
